# Rebooting artificial intelligence for health

**DOI:** 10.1371/journal.pgph.0004171

**Published:** 2025-01-17

**Authors:** William Greig Mitchell, Judy Gichoya Wawira, Leo Anthony Celi

**Affiliations:** 1 Department of Ophthalmology, Royal Victorian Eye and Ear Hospital, Melbourne, Australia; 2 Centre for Eye Research Australia, Melbourne, Australia; 3 Department of Radiology and Biomedical Informatics, Emory University, Atlanta, GeorgiaUnited States of America; 4 Institute for Medical Engineering and Science, Massachusetts Institute of Technology, Boston, Massachusetts, United States of America; 5 Department of Biostatistics, Harvard TH Chan School of Public Health, Boston, Massachusetts, United States of America; 6 Department of Medicine, Beth Israel Deaconess Medical Centre, Boston, Massachusetts, United States of America; PLOS: Public Library of Science, UNITED STATES OF AMERICA

The rapid, unregulated integration of Artificial Intelligence (AI) into clinical workflow has raised concerns among academic researchers. During the COVID-19 pandemic, Epic introduced a tool to predict early sepsis, marketed as ‘product enhancement’ to evade regulatory approval. This tool was later withdrawn after external validation revealed it missed 67% of sepsis cases, and had an area under the curve of only 0.63, significantly lower than initially reported [1]. Another clinical deterioration prediction tool from Epic was withdrawn during the pandemic after it was shown to be far less accurate than claimed. It was reworked and re-released but continued to perform poorly in a recent study [2]. Similar prediction tools introduced into clinical practice have also failed when evaluated in diverse populations, necessitating withdrawal [3]. Today, digital scribes are being widely adopted in clinics to reduce the administrative burden of notetaking, despite being originally designed to summarize non-medical text. These tools, too, lack sufficient data on their utility, validity, and impact on patient safety [4]. Exploiting loopholes in regulations has allowed these tools to be integrated into healthcare largely without oversight.

One core issue is that AI systems are trained on historical data reflective of existing structural inequalities. Most datasets are derived from patients in high-income countries (HICs), with underrepresentation of low- and middle-income countries (LMICs) and marginalized groups who stand to benefit most. While developing technological infrastructure in LMICs is important for long-term data equity, diligent external validation and recalibration of algorithms trained on HIC data in global settings is crucial in ensuring clinical AI is meaningful for broader populations [5]. Additionally, available data is influenced by biases inherent in clinical decision-making. For example, a female patient may face a higher mortality risk after an acute coronary event when treated by a male cardiologist given the known effect of gender discordance on outcomes after a heart attack [6]. Or a Black patient may have arterial oxygen saturation readings that are inaccurately normal due to flaws in fingertip oximetry for more pigmented patients [7]. If we train models on data without understanding the context in which it was collected, AI learns spurious erroneous associations, such as ‘being a woman is a risk factor for poor outcomes after a heart attack’ (rather than being a woman cared for by male cardiologists who outnumber female cardiologists), and ‘being Black is a risk factor for poor outcomes from pneumonia’ (rather than being undertreated with oxygen because of erroneous fingertip oximetry reading). These learned spurious associations are encrypted in algorithms with unpredictable downstream effect on patient outcomes.

There are inadequate safeguards throughout the AI development pipeline. While more robust regulatory frameworks are essential, we must take collective responsibility for the fair development of AI at every stage of its lifecycle. This includes ensuring rigorous checkpoints from initial data collection, aggregation, standardization and curation, to algorithm development, evaluation, and external validation [8]. Assessing clinical utility rather than statistical model performance and conducting real world monitoring are critical. These are currently not and unlikely to be mandated, with no incentives for health systems to perform this continuous evaluation of impact on patient outcomes after AI deployment. Such assessment of commercial models is challenging, where the algorithm is proprietary. Subsequently, methods to dissect how the data came about, how the models were developed and by whom and metrics that benchmark performance against clinical effectiveness and seamless workflow integration are required to predict performance between populations and across clinical practice patterns [9]. Acknowledging the dynamic nature of AI development (which is in tension with the static nature of algorithm approval), it is crucial to involve patients, especially those from marginalized communities, in developing and evaluating AI systems to get feedback on what is valuable across groups. At each step of the model development and deployment process, we must double check the value of the model across a diverse group of users (i.e., “does this task need AI?”), deliberately seek the requisite perspectives to make this determination, and repeatedly verify the assumptions made during data harmonization, curation and analysis.

Moreover, we need to prioritize transparency regarding how data is used, going beyond a narrow definition of data ‘privacy’ [10]. This is critical for two reasons – firstly, because the rapid rise of generative models demands more and more data; and secondly, due to the growing interest in developing multimodal algorithms. These data require more detailed variables, increasing the risk to patient re-identification. Auditing models is challenging when multiple datasets are combined, necessitating linkage to source data. In the era of foundation models, dataset derivatives including embeddings and synthetic data, or subsets of datasets like masks are not human-interpretable, yet encode significant patient characteristics. Finally, it is common to deviate from the original task a dataset was intended for – and ethics review boards cannot anticipate or evaluate these pivots. There is urgency in the need to rethink data provenance and establish clear oversight of data usage. In most of these dialogues, the patient voice is missing, and we must provide the platform to empower and equip patients to understand how their health data are utilized and monetized.

To borrow an analogy from *The Matrix*, we face a choice: the blue pill represents complacently accepting the limitations of current AI systems, while the red pill symbolizes awakening to these realities and reimagining the future of AI through humility and diversity. Paradoxically, AI has crystalized the necessary steps forward. By aggregating complex data sourced from disparate contexts, it challenges us to prioritize interdisciplinary collaboration–bringing together computer scientists, clinicians, public and global health specialists and social scientists from all over the world to address the root causes of healthcare disparities. This also means broadening collaborations with leaders from historically underrepresented populations, like LMICs, the Global South, and Black, Indigenous and people of colour (BIPOC) experts, and continuing to tackle parachute research [11,12]. The complexity of healthcare data necessitates this collective approach.

The potential for AI to drive systemic change in healthcare is immense. However, if we do not address the challenges it presents, we risk deepening divides, reinforcing groupthink, and perpetuating disparities. This is the true existential risk of AI, not the spectre of sentient machines. Models must be developed with epistemic humility and diversity, assessing their clinical utility *with* those who stand to benefit/suffer from their predictions before deployment. Their real-world performance must be continuously monitored, with anticipation that their effect on user behaviour, and consequently clinical outcomes, will evolve and diverge across patient demographics, geographies, and practice settings. Algorithms used for commercial models must be publicly available to interrogate, rather than understanding their predictions via proxy. And most of all there must be transparency in how patient data is utilized and monetized.

There is hope in the growing commitment of young people, activists, and scientists to tackle these systemic issues. This, ultimately, is the true legacy of AI.

## References

[1] Habib A, Lin A, Grant R. The epic sepsis model falls short–the importance of external validation. JAMA Intern Med. 2021;181(8):1040-1041. doi: 10.1001/jamainternmed.2021.3333 34152360

[2] Edelson DP, Churpek MM, Carey KA, Lin Z, Huang C, Siner JM, et al. Early warning scores with and without artificial intelligence. JAMA Netw Open. 2024;7(10):e2438986. doi: 10.1001/jamanetworkopen.2024.38986 39405061 PMC11544488

[3] Byrd TF, Southwell B, Ravishankar A, Tran T, Kc A, Phelan T, et al. Validation of a proprietary deterioration index model and performance in hospitalized adults. JAMA Netw Open. 2023;6(7):e2324176. doi: 10.1001/jamanetworkopen.2023.24176 37486632 PMC10366696

[4] Buchem MMv, Boosman H, Bauer MP, Kant IMJ, Cammel SA, Steyerberg EW, et al. The digital scribe in clinical practice: a scoping review andresearch agenda. NPJ Digital Medicine. 2021;4(1)57. 33772070 10.1038/s41746-021-00432-5 PMC7997964

[5] Celi LA, Cellini J, Charpignon M-L, Dee EC, Dernoncourt F, Eber R, et al. Sources of bias in artificial intelligence that perpetuate healthcare disparities-a global review. PLOS Digit Health. 2022;1(3):e0000022. doi: 10.1371/journal.pdig.0000022 36812532 PMC9931338

[6] Greenwood BN, Carnahan S, Huang L. Patient-physician gender concordance and increased mortality among female heart attack patients. Proc Natl Acad Sci U S A. 2018;115(34):8569–74. doi: 10.1073/pnas.1800097115 30082406 PMC6112736

[7] Sudat SEK, Wesson P, Rhoads KF, Brown S, Aboelata N, Pressman AR, et al. Racial disparities in pulse oximeter device inaccuracy and estimated clinical impact on COVID-19 treatment course. Am J Epidemiol. 2023;192(5):703–13. doi: 10.1093/aje/kwac164 36173743 PMC9619495

[8] Futoma J, Simons M, Panch T, Doshi-Velez F, Celi LA. The myth of generalisability in clinical research and machine learning in health care. Lancet Digit Health. 2020;2(9):e489–92. doi: 10.1016/S2589-7500(20)30186-2 32864600 PMC7444947

[9] Obermeyer Z, Powers B, Vogeli C, Mullainathan S. Dissecting racial bias in an algorithm used to manage the health of populations. Science. 2019;366(6464):447–53. doi: 10.1126/science.aax2342 31649194

[10] Savage N. Privacy: the myth of anonymity. Nature. 2016;537(7619):S70–2. doi: 10.1038/537S70a 27602747

[11] Robinson J, Kyobutungi C, Nyakoojo Z, Pai M. Editors as allies: our two-year experience at PLOS Global Public Health. PLOS Glob Public Health. 2023;3(11):e0002644. doi: 10.1371/journal.pgph.0002644 38011250 PMC10681289

[12] The Lancet Global H. Closing the door on parachutes and parasites. Lancet Glob Health. 2018;6(6):e593. doi: 10.1016/S2214-109X(18)30239-0 29773111

